# Reflections on the past two decades of Mind, Brain, and Education

**DOI:** 10.1111/mbe.12407

**Published:** 2024-03-02

**Authors:** Ola Ozernov-Palchik, Courtney Pollack, Elizabeth Bonawitz, Joanna A. Christodoulou, Nadine Gaab, John D.E. Gabrieli, Patricia Monticello Kievlan, Christina Kirby, Grace Lin, Gigi Luk, Charles A. Nelson

**Affiliations:** 1 Harvard Graduate School of Education; 2 McGovern Institute for Brain Research, MIT; 3 MGH Institute of Health Professions; 4 Prelude Music Foundation; 5 Porticus Foundation; 6 Department of Psychology, Harvard University; 7 Scheller Teacher Education Program The Education Arcade, MIT; 8 Department of Educational and Counselling Psychology, McGill University; 9 Department of Pediatrics, Harvard Medical School; 10 Division of Developmental Medicine, Department of Pediatrics, Boston Children’s Hospital

In the early 2000s, Kurt Fischer and colleagues founded the Mind, Brain, and Education (MBE) field (Blake & Gardner, 2007; Fischer & Bidell, 2006), including a flagship journal, society (International Mind, Brain, Education Society [IMBES]), and a master’s degree program at the Harvard Graduate School of Education (Harvard). The MBE program was the first-of-its-kind, focused on the intersection of neurobiology, psychology, and educational research and practice (Blake & Gardner, 2007; Fischer, 2009). Between its first cohort in 2004 and its final cohort in 2022, the program graduated 668 students from around the world (see Figure 1). Contemporaneously, scholars developed MBE or related Educational Neuroscience initiatives in several US states, Canada, the United Kingdom, Austria, The Netherlands, China, Israel, across Latin America, and other locations around the world.

In 2022, the MBE masters program was integrated into a broader human development and education offering at Harvard. Program faculty and alumni gathered in a virtual event to mark the sunsetting of the program and to reflect on advancements in the MBE field. This commentary grew out of that meeting and reflects the perspectives of individuals affiliated with MBE on the evolution and impact of the field, with an eye toward future directions. Specifically, to guide their reflections (for contributing authors, see Figure 2), all authors addressed the following questions:

1. How has the field of MBE changed, and what developments particularly excite you in your area of the field?
2. What do you regard as the most significant impact of MBE on educational practice?
3. As you look ahead, what potential advancements or emerging trends do you envision for MBE research and its practical applications?

## OLA OZERNOV-PALCHIK

A central question in education revolves around what instructional approaches work for whom and under which conditions. MBE seeks to address these inquiries by lever-aging cross-disciplinary methods and frameworks with rigorous scientific precision. Yet, the progression of scientific knowledge is often limited by the tools at hand. Herein lies one of the most significant contributions of MBE: It acts as a catalyst for methodological innovation, highlighting the limitations of current scientific approaches in tackling some of the most pressing educational challenges.

The need to model the complex probabilistic relations that underlie educational response propelled innovation in the application of sophisticated computational techniques to highly dimensional student data. To illustrate, in a recent study, we used machine learning (ML) to improve methods of prediction of instructional responses in first-grade students at risk for reading disabilities (Shangguan et al., Under Review). Due to challenges such as incomplete data from young participants, our initial ML models struggled to forecast student outcomes accurately. It wasn’t until we integrated methods adapted from the fields of natural language processing and computer vision that we saw a significant uptick in accuracy. We trained our models to recognize hidden patterns between known and unknown data points, resulting in an enhanced out-of-sample prediction accuracy of 80%. In another example, innovative ML was applied to video recordings of math lessons to identify what teacher discourse features were important for supporting a positive learning mindset in students (Hunkins, Kelly, & D’Mello, 2022).

In a different domain, the need for more ecologically valid neuroimaging to measure how cognitive and neural processes that underlie learning unfold in naturalistic settings, has facilitated the optimization of portable neuroimaging methods across a diverse range of contexts. For example, portable electroencephalography has been used to study student engagement during live classroom instruction (Davidesco, Matuk, Bevilacqua, Poeppel, & Dikker, 2021; Landi et al., 2019), and functional near-infrared spectroscopy has been used to document neural signatures of reading development in children growing in environments with a high risk of illiteracy, rural Côte d’Ivoire (Jasińska & Guei, 2018). The implementation of portable neuroimaging in these settings posed methodological challenges, and the response to those challenges fostered innovation. In-school EEG data collection is noisy, so the team has leveraged high-density EEG tools, which capture thousands of data points per second, combined with ML classifiers, to dissociate signal from noise. This allowed for a more precise characterization of individual differences in the neural substrates of reading as they unfold during instruction (Davidesco et al., 2021; Landi et al., 2019). To address the challenges of setting up a portable neuroimaging laboratory in low-resource contexts, researchers developed comprehensive protocols addressing problems such as high humidity and lack of Internet access (Jasińska & Guei, 2018). The advancement of these portable neuroimaging methods and the emergence of additional technologies, such as portable magnetic resonance imaging, can enable more equitable access to neuroimaging technologies around the world.

## COURTNEY POLLACK

Over the past 20 years, the MBE field has facilitated the expansion of empirical, translational, and policy-relevant work through a breadth of opportunities to bridge the mind, brain, and education sciences. Using mostly lab-based cognitive neuroscience approaches (but see Janssen et al., 2021), educational neuroscience researchers have examined the cognitive and neural bases of school-relevant skills. Not meant to impact the classroom directly, this research has elucidated the neurocognitive mechanisms that underlie learning, such as basic processes in numerical cognition (Pollack & Price, 2019, 2020), the relation between math and reading (Pollack et al., 2021; Pollack & Ashby, 2018), and the connection of both with executive functioning (Marks et al., 2023). Connecting to classroom learning, MBE work has advanced understandings of education challenges across levels of analysis. For example, MBE work has evidenced the deleterious biological and psychological effects of racial/ethnic discrimination and how they undermine student performance and opportunities to thrive (Levy, Heissel, Richeson, & Adam, 2016; Shonkoff, Slopen, & Williams, 2021), and translated research for myriad education stakeholders (The Learning Scientists, 2023; https://bold.expert/). MBE’s interdisciplinary approach has also informed policy. A synthesis of convergent evidence from biology, psychology, and education shows that adolescents benefit from starting school later (Watson et al., 2017), which provided strong evidence for California’s 2022 implementation of later middle and high school start times (SB-328 Pupil Attendance: School Start Time, 2019).

Alongside the expansion of MBE research, additional training and career development opportunities support the field’s expansion, including the 2017 establishment of the IMBES Trainee Board by early career scholars (Gotlieb et al., 2019). The international proliferation of programs and centers (e.g., University of Alabama, Vanderbilt University, Vrije Universiteit Amsterdam, University of Graz, East China Normal University, University College London) has made joining the community easier.

I am particularly enthusiastic about the field’s potential moving forward. MBE stakeholders can expand research-practice partnership opportunities, emphasize learning from educational practice, and further increase access and representation in the questions we ask and who may answer them.

## ELIZABETH BONAWITZ

Educators have long been aware of a simple fact: sparking a child’s curiosity will help them learn (Deci & Ryan, 1981). Developmental psychologists have suggested that children’s insatiable wonder is a critical drive in cognition that supports intuitive theory building and scientific reasoning (Gopnik, 2016; Keil, 2022; Schulz, 2012). Robotics, ML, and artificial general intelligence researchers have begun to build models with “curiosity” rewards to bootstrap learning (see Haber, 2022 for recent review). Psychological and computational theories link it to our need to resolve uncertainty and fill knowledge gaps (Gottlieb, Oudeyer, Lopes, & Baranes, 2013; Loewenstein, 1994). But until recently, formalizing exactly what curiosity is and how it relates directly to learning and the brain has remained less well understood (Kidd & Hayden, 2015).

One of the most exciting developments in MBE has been new brain measures that might “tap into” and even provide explanation for the function and mechanisms underlying our drive to resolve uncertainty. In particular, EEG studies (measuring the electrical signals in the brain) have revealed several important connections between the theta oscillation brain signal and behavior: theta oscillations are involved in memory formation and reward; they are predictive of learning success; and, even in infancy, they are heightened preceding events that are predicted to reveal new information (see Begus & Bonawitz, 2020 for review). Such measures allow us to decouple previously confounded measures of curiosity and learning, such as the child’s exploration (which can be influenced by factors beyond curiosity) and information gathered (which affects the inferences that can be drawn). We can then also understand how the child’s current beliefs and preceding experiences (e.g., established knowledge, goals, and rapport with a teacher, see Bonawitz & Shafto, 2016) affect a learner’s expectation of information and curiosity. And we can study more precisely how brain measures that might reflect curiosity then relate to attention, memory, and learning. Such explanatory models will allow us to understand better how to support curiosity in all learners. It is a very exciting time, as we are just at the precipice of building causal, theory-driven models of the role of curiosity in the mind and brain for education.

## JOANNA A. CHRISTODOULOU

Since the early 2000s, the field of MBE has continued to provide novel insights and perspectives across research-practice-clinic settings. The power of this research has been showcased in how we conceptualize learning differences, reading acquisition, and brain plasticity. With a focus on learning disabilities or difficulties, I draw on the example of dyslexia, a common reading disability that impacts the accuracy and/or fluency of single-word reading (Lyon et al., 2001; Lyon et al., 2003).

One of the earliest contributions of cognitive neuroscience research was in establishing that dyslexia was indeed “neurobiological in origin,” in that brain signatures for reading differ between groups of readers with and without dyslexia. Although seemingly elemental, this insight continues to act as a framework essential in reframing attribution for the difficulties faced by struggling readers (i.e., it was not based on laziness, limited aptitude, etc. as many continue to believe; Cortiella & Horowitz, 2014; Horowitz, Rawe, & Whittaker, 2017). Furthermore, the accumulated evidence that the “reading brain” is plastic, and changes in ways specific to the reading programs used (Eden et al., 2004; Yoncheva, Wise, & McCandliss, 2015), has redoubled the importance of providing evidence-based reading instruction to developing readers. MBE research has also converged with theories of learning (e.g., dynamic skills theory; Fischer & Bidell, 2006) to reveal that skill acquisition is dynamic, with the impact of reading instruction differing by student and environmental characteristics, as well as by academic calendar phases (i.e., during summer vacation; Romeo et al., 2018; Meisler et al., 2023). Insights from studies on reading abilities among individuals with distinct neural architecture (e.g., relying on a single hemisphere as a consequence of epilepsy-related surgery) have likewise emphasized the resilience of students and the multiple pathways to reading progress (Christodoulou et al., 2021; Katzir, Christodoulou, & DeBode, 2017). Across these types of contributions, MBE has advanced how we conceptualize learning and teaching.

Looking ahead, MBE can harness the vast foundational knowledge now established to explore more complex and nuanced studies of children in contexts (e.g., examining the interactions of who a child is with what is being asked and in what context). Another potential for innovation is advancing toward MBE research that shifts away from group-based inquiries to individual differences models. Most importantly, MBE research will matter in the future when it is disseminated effectively, in a manner inclusive and representative of our populations of interest, and meaningful for the communities we serve as scientists.

## NADINE GAAB

When the MBE field started to emerge, it had a number of bold ideas and hypotheses. For example, people were excited about the prospect of using neuroimaging to diagnose learning differences, characterize individual learning trajectories, or as a tool to identify who will subsequently struggle with learning to read or math. Furthermore, there was excitement about a direct translation of neuroimaging findings into the classroom using “brain-based curricula.” Over the last 20 years, the field has learned a lot about neuroimaging methodology and its limitations and has also started to ask more refined questions and build relationships among the various stakeholders in the scientific and educational settings. As a result, evidence-based translational knowledge and scientifically trained change-makers have emerged (Solari et al., 2020; Ansari & Coch, 2006; Sheridan & McLaughlin, 2022; Thomas et al., 2019; Blakemore & Bunge, 2012; Gabrieli, 2016). In my opinion, the most significant impact of MBE on educational practice is at least twofold. First, we now understand that the development of academic skills and their precursors starts as early as in utero. Therefore, we need to understand a child’s entire developmental trajectory (both generally and in key skills such as language and reading) in the context of variable environments to determine how, when, and where brains learn best (Ozernov-Palchik, Yu, Wang, & Gaab, 2016). Second, inspired by neuroscientific evidence that brain differences underlying disorders such as developmental dyslexia are present as early as infancy (Guttorm et al., 2005; Langer et al., 2017), the idea of “preventative education” is emerging, and early identification of children at risk for atypical developmental trajectories of academic skills is starting to get embraced (Fletcher et al., 2021; Gaab & Petscher, 2022; Catts & Petscher, 2022; Catts & Hogan, 2021; Ozernov-Palchik et al., 2016). All but 11 states in the United States have passed legislation for universal kindergarten literacy screening designed to identify children at risk for literacy-based disabilities (https://improvingliteracy.org/state-of-dyslexia). Education needs to continue to move from a reactive model to a proactive model, similar to shifts that happened in other fields that led to impactful practices such as preventative medicine (e.g., mammograms for breast cancer detection, Duffy et al., 2020).

## JOHN D. E. GABRIELI

The field of MBE began with great excitement as modern neuroimaging techniques allowed for visualization and measurement of the brain bases of learning, how that develops from infancy through adulthood, and how that varies in different kinds of learners. It was, and is, thrilling to view the functional and structural brain bases of learning, the essence of education. In broad terms, MBE embraced the idea that, where possible, understanding and improving educational outcomes needs to be fueled by scientific evidence. For centuries, the understanding of the brain bases of learning was informed only by the study of adults with focal brain injuries. Now a neuroscience perspective could be brought directly to children and their developing brains, and to many aspects of education and learning, from reading to math and to broader cognitive skills, like executive functions, and social-emotional functions that support all kinds of learning. A change over time is that neuroimaging research has broadened its perspective from a focus on specific learning differences to consider also differences related to environmental factors, such as socioeconomic status, that have such broad impacts on education outcomes and opportunities (Mackey et al., 2015).

One significant contribution of MBE was that powerful evidence became available for the brain bases of learning differences, such as dyslexia. Diversity among children’s brains can make various aspects of learning to read easier or harder. Indeed, such differences are now documented for risk for dyslexia from infancy (Yu et al., 2022) through the start of school (Saygin et al., 2013) and for dyslexia through the years of schooling into adulthood. Further, the identification of differences in pre-readers opened up opportunities for early support that prevent severe struggles.

Neuroimaging also revealed brain plasticity associated with helpful educational practices, which further supported the importance of such practices. In some cases, neuroimaging outperformed typical education measures in predicting future improvement in reading among children with dyslexia (Hoeft et al., 2011). The idea that brain measures, in combination with typical education measures, could help identify which student is most likely to benefit from alternative supports opens the possibility of more effective individualized education.

## PATRICIA MONTICELLO KIEVLAN

The MBE field has had a profound and positive impact on education for neurodiverse students and their families. A learning difference diagnosis can describe a student’s challenges, but it can also be perceived as overly prescriptive, limiting the potential for a student’s future academic success. Neuroimaging studies have revealed that children with learning differences recruit alternative pathways and develop compensatory strategies to achieve success in the domain in which they are impaired (Yu et al., 2020; Zhao et al., 2023). For students with diagnoses of attention deficit hyperactivity disorder or dyslexia, this idea that learning involves many concurrent processes is empowering: Even if a student faces some challenges, they can rely on other strengths and capabilities to find success.

In my work as an educator, I have used the MBE approach as a tool for combating misleading claims about learning and the brain. As a music teacher, I have used my MBE background to help parents overcome misleading information about music and child development (like the so-called “Mozart effect”) and instead provide more reliable insights about how early music exposure can help young children’s developing speech and language (Tierney & Kraus, 2013; Zhao & Kuhl, 2016). With its focus on applying scientific evidence to inform educational practice, MBE helps educators to be more discerning consumers and effective stewards of information about learning and the brain.

## CHRISTINA KIRBY

As an education funder for a global philanthropic organization, Porticus (2023), I have the privilege of learning from many nonprofit leaders, practitioners, public policymakers, and the like. From this bird’s-eye view, I’ve witnessed a shifting interest in incorporating the learning and developmental sciences—the crux of MBE—into practice and policy. Perhaps the most revolutionizing contribution from MBE to the education sector has been an evidence-based understanding of the conditions required to support learning. Organizations like Turnaround for Children (https://turnaroundusa.org) convey the clear science that all children are born with brains that are ready to learn, that learning depends on conditions in the environment, and that we must design care and educational settings that enable children to thrive (Jensen et al., 2021; Troller-Renfree et al., 2022; Troller-Renfree et al., 2023). A sense of belonging, safe environment, supportive relationships, and a holistic approach to building student skills, mindsets, and autonomy are fundamental to learning (Caballero et al., 2019). The resources published by networks such as the Science of Learning and Development Alliance (2023) are critical to translating these scientific principles into actionable guides for implementation, like the Design Principles (Learning Policy Institute & Turnaround for Children, 2021).

What excites me most is seeing these tenets being implemented in pockets across the globe. NGOs have been thought leaders in bridging the knowledge-practice gap. For instance, in addition to Turnaround, Transcend Education (https://transcendeducation.org) uses a community-driven R&D model to build school environments with evidence-based design and practices. These organizations and others are working with districts across the country, such as Washington DC Public Schools, to redesign systems with contextualized MBE principles embedded within their core operations.

## GRACE LIN

MBE has made a lasting contribution to education with the research-supported Universal Design for Learning framework (UDL; Rose & Meyer, 2002). UDL emphasizes diverse populations; it offers actionable guidelines for enacting change and the optimal design of educational experiences for all. UDL has permeated education practice and policy (e.g., Higher Education Opportunity Act, 2008; Individuals With Disabilities Education Act, 2004) and influenced the design and implementation of educational materials, including curricula for Artificial Intelligence (AI) education (Walsh et al., 2022).

The AI for K-12 initiative (AI4K12) highlights considerations for AI technology in education, such as biases in data that AI models use for training, issues of equity in AI, and societal impacts (Touretzky, Gardner-McCune, Martin, & Seehorn, 2019). In response, designers, researchers, and stakeholders use the UDL framework to make curricular materials accessible and engaging (Walsh & Dalton, 2022). In codesigning learning activities for an inclusive AI literacy curriculum, Responsible AI for Computational Action (https://raise.mit.edu/research-projects/inclusiveai-literacy-learning/), I witnessed special needs teachers on the research team and field-based teacher partners cite UDL in their curriculum modifications, for example, the UDL principle of multiple modes of expression. Teacher partners modified a module in which students redesign a space and incorporate AI technology in an all-digital format to allow students to build physical miniature models of their envisioned room, which would help students articulate their designs and thoughts. UDL’s translation from the research base to practical, actionable guidelines can facilitate the wide adoption of brain-science-informed learning design decisions among educators.

As MBE evolves, we should celebrate its progress and continue to expand its mainstream adoption through the translation of cutting-edge research into practical applications and frameworks. Such translation can facilitate practitioners’ and designers’ uptake of these advancements, ultimately enhancing the inclusivity and equity of educational programs.

## GIGI LUK

In the last few years, I have observed a growing interest among educators (in North America and beyond) seeking knowledge on child development through cognitive neuroscience, focusing on mechanisms underlying behavioral outcomes. From early childhood education to college learning, schools often become children and adolescents’ “second home” where they spend an extended amount of time. Educators and schools are active agents in shaping not only children’s learning outcomes, but also their development. I had the pleasure of working with several educators who actively contributed to informing and designing our experiments. Furthermore, many of the doctoral students I worked with at the Harvard Graduate School of Education (HGSE) were teachers and educators, creating synergetic collaborations merging their professional experiences with research.

Personally, as a researcher engaging in both cognitive neuroscience and education research relating to multilingualism, embracing the MBE approach has changed me to be a better learner and listener. By spending time talking to educators at the state-, district-, and school-levels as a cognitive neuroscience researcher of language, I learned about their challenges and achievements from practitioner and policymaking perspectives. By observing Head Start classrooms and interacting with parents and educators, I listened to their concerns and gained a grounded perspective on child development. These voices enriched the way my research is framed and designed. For example, the shift from viewing multilingualism as a homogeneous group in state-level data (Luk & Kroll, 2019; Mancilla-Martinez, Oh, Luk, & Rollins, 2022; Yamasaki & Luk, 2018), and the shift to focus on outcomes to process and individual language experiences (Leon Guerrero, Whitford, Mesite, & Luk, 2021; Luk, 2022; Smith, Leon Guerrero, Surrain, & Luk, 2021) facilitate the understanding of learning mechanisms, which informs pedagogical strategies in linguistically diverse classrooms. I see the next stage of MBE being a two-way street, bringing researchers and stakeholders of education together, centering on child development and learning, and synergistically enriching scholarly knowledge, pedagogical practice, and educational policy. MBE-minded researchers, practitioners, and policymakers situated in different contexts aim to improve children’s well-being by investing in MBE collaborations and synergy to transform cognitive neuroscience to be ecologically meaningful and relevant.

## CHARLES A. NELSON III

Over the past two decades, I have witnessed an eagerness among educators to learn more about the brain and brain development and to explore how knowledge of brain development can inform the field of education. Embedded within this is a curiosity about how one studies human brain development and thus, an interest in learning more about the broad field of neuroimaging.

In terms of what most excites me, several things come to mind. First, there have been major advances in our understanding of errors in brain development that lead to children struggling to learn to read (dyslexia; Norton, Beach, & Gabrieli, 2015), to master mathematics (dyscalculia; De Smedt, Peters, & Ghesquière, 2019), and to communicate socially (autism; Willsey & State, 2015). These errors can be genetic (e.g., single gene disorders such as Rett Syndrome or Fragile X Syndrome) or environmental (e.g., children experiencing profound neglect early in life, such as those abandoned in orphanages McLaughlin, Sheridan, & Nelson, 2017). For example, in one study, my colleagues and I demonstrated the causal effects of the caregiving environment on children’s stress response during academic-like tasks (e.g., mental arithmetic; McLaughlin et al., 2015). Second, advances in neuroimaging have increasingly allowed us to identify children at risk for developmental disorders very early in life (e.g., work on the development of biomarkers that can be used as objective “readouts” of atypical development, before behavioral readouts become reliable). For example, across multiple studies, we found that atypicalities in brain function in infants can facilitate earlier identification of autism spectrum disorder (Bosl, Tager-Flusberg, & Nelson, 2018; Peck et al., 2021).

## CONCLUSION

MBE as a field has evolved considerably, and there continues to be a lot to be excited about! There have been substantial advancements in mechanistic descriptions of typical and disordered development across domains such as reading, mathematics, creativity, and social communication. Furthermore, MBE research has generated complementary neurobiological evidence in support of educational policies and practices, such as preventative universal literacy screening and a later school start time, to optimize learning and healthy development across the life span. While MBE has achieved notable progress in its two-decade existence, it continues to strive to fulfill its foundational promise of enhancing precision in risk identification, outcome prediction, and individualized education (Gabrieli, 2016). In closing this paper, we distill the insights from the individual contributions into three main challenges hindering MBE’s influence on educational practice and policy: (1) inter-individual heterogeneity, (2) limited generalizability, and (3) gaps in the implementation of scientific evidence. We include some hopeful developments for addressing these challenges.

For neuroscience to advance education, it is critical to understand how the nervous system relates to behavior by examining the associations between measures of the brain and variation in some behavior. Measures of brain and behavior, however, are complex, with multiple factors that influence their reliability (Elliott et al., 2020, 2021; Noble, Scheinost, & Constable, 2019; Zorowitz & Niv, 2023). Consequently, brain-behavior associations yield very small effect sizes and require enormous sample sizes (in the thousands!) to detect these effects reliably (Marek et al., 2022). To address issues of optimizing sample sizes and increasing reliability, the field has been moving toward multi-lab collaborations, resulting in consortia with thousands of participants (e.g., Adolescent Brain Cognitive Development Study; Volkow et al., 2018) and in-depth multi-session characterization of individuals using precision neuroimaging approaches (Grotton et al., 2022). These approaches will enable a comprehensive understanding of how the brain is related to behavior.

The application of research findings across populations is critical for the advancement of knowledge, and in turn for the application of findings to diverse communities. As evident in the examples highlighted in the current paper, most MBE studies to date have been conducted in non-naturalistic lab settings, under tightly controlled conditions, and on non-representative WEIRD (Western, Educated, Industrial, Rich, and Democratic; Henrich et al., 2010) populations. There has been a shift toward increased focus on engaging and training diverse communities in research and transitioning research from labs to schools and communities. Such efforts are necessary to establish the boundaries of generalizability of findings and determine what is true for what populations.

Finally, the adoption of evidence-based practices in education has been impeded, in part, by a paucity of knowledge regarding factors that affect their implementation and sustainability in educational settings. Currently, there is an intensified focus on pinpointing the components of specific programs, discerning the barriers and facilitators across various educational contexts, understanding the structural characteristics of the participating institutions, and evaluating the individual factors of those targeted and those implementing the program, crucial for successful implementation and sustainability. Drawing from this work, the field has increasingly emphasized engagement in equitable and sustainable research collaborations with educators.

## Figures and Tables

**Fig. 1.**
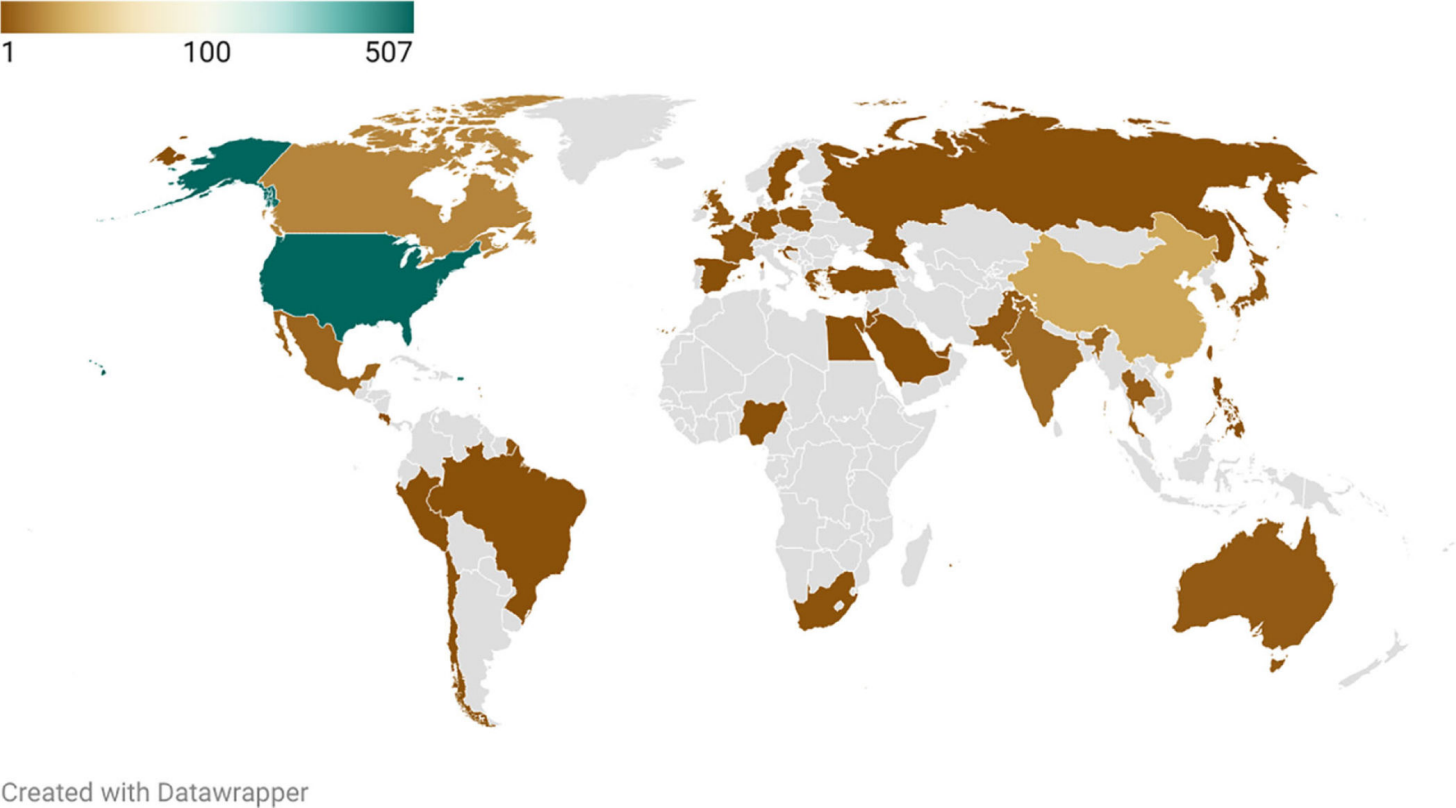
Map of the number of graduates of the Mind, Brain, and Education program at Harvard between 2004 and 2022, by country (N = 668). Data courtesy of Harvard Graduate School of Education.

**Fig. 2.**
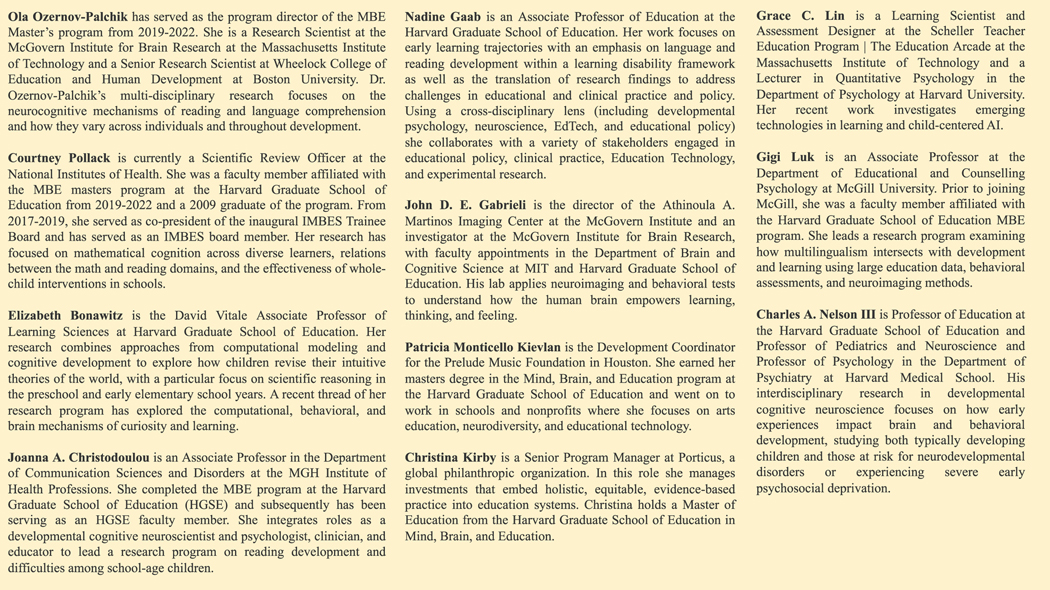
The contributors.
